# Obstructions within the Upper Respiratory Tract of Children; Their Relation to General Health

**Published:** 1895-08

**Authors:** Henry J. Mulford

**Affiliations:** Buffalo, N. Y., Clinical Instructor in diseases of the nose and throat, Medical Department, University of Buffalo.


### OBSTRUCTIONS WITHIN THE UPPER RESPIRATORY TRACT OF CHILDREN; THEIR RELATION TO GENERAL HEALTH.

By HENRY J. MULFORD, M. D., Buffalo, N. Y.,

Clinical Instructor in diseases of the nose and throat, Medical Department, University of Buffalo.

THE physician is the first event in the child's life. It is his duty to see that the new life begins without embarrassment; his duty to watch that no embarrassment can occur. Periodical examination of the child must be insisted upon. At the very hour of birth, trained inspection should be begun. Many faults and ills might be avoided if there were inspection at regular intervals from birth to adult life.

Within the upper respiratory tract of the child lurks a constant source of danger. It is here tissue overgrowth does most harm; the harm of slow obstruction and of long-standing irritation. It gives no fear of sudden death; this blocking of life's gateway causes worse than death—stupidity. So familiar are these conditions of obstruction, that we dismiss them as trivial. But to their presence is due the failure of many a life. In a situation easy of access they are recognised without difficulty; only carelessness avoids them. One glance is enough, the face itself tells the story. In long-standing obstruction it is the face of dull stupidity—the physiognomy of pressure.

The commonest conditions giving danger to the child arise from overgrowth of tissue within the vault, and hypertrophy of tonsils within the pharynx. They work harm in three ways: by obstructing respiratory currents; by diverting respiratory currents into the wrong channel; and, reflexly. To illustrate, here is presented a limited number of cases showing conditions leading to the above results.

CASE I.—November, 1894. E. S., boy, aged 6 years; measles three years ago; tonsils large since; lately very large. Has nightmare, very nervous, no appetite. Dark circles under eyes, extreme anemia; mouth breather. Removed both tonsils under chloroform; hemorrhage slight, recovery prompt. Administered liquor Dobell locally and syr. ferri iodid. internally. Ten days after, reported marked improvement in general condition. At this writing none of above symptoms are present.

CASE II.—May, 1893. L. G., boy, aged 9 years; sore throat and cough for several weeks; more or less trouble with throat all the time, nose occluded. Very anemic, weak; appeared delicate. Hypertrophy of both tonsils; hypertrophy of tissue within pharyngeal vault; pigeon breast. Removed all hypertrophied tissue under chloroform; no hemorrhage; recovery good, but slow. Administered liquor Dobell locally and syr. ferri iodid. internally. An acute pharyngitis followed operation; after one week, did well. Rapid return to strength and health.

CASE III.—March, 1895. T., girl, aged 18 years; throat trouble for more than a year; frequent sore throat; frontal headache; constant cough; appetite poor. Both tonsils greatly hypertrophied. Tonsils removed without anesthetic; sharp arterial hemorrhage for ten minutes. Administered locally the following: Acid tannici, gr. xx.; liquor Dobell, f℥iv.; locally. Four days after operation, seat of operation acutely inflamed; responded to treatment readily. One month after operation cough and headache had disappeared; appetite returned.

CASE IV.—June, 1894. M. H., girl, aged 5 years; frequent sore throat and earache; mouth breather. Pharyngeal vault small, filled with hypertrophied tissue. Removed under chloroform and ether; no hemorrhage. Used liquor Dobell locally. Recovery good. No more earache.

CASE V.—March, 1895. J. M., girl, aged 7 years; scarlet fever last Summer; since then throat trouble. Nervous, sleeps poorly, with mouth open. Tall and poorly nourished. Both tonsils and tissue of vault hypertrophied. All removed under chloroform; no hemorrhage; recovery good. Regained normal condition rapidly.

CASE VI.—January, 1895. S. B., girl, aged 11 years; takes cold easily; frequent sore throat, nose occluded. Mouth breather, tall, anemic, nervous, restless during sleep. Tonsils very large; vault well filled. Removed all under chloroform; slight hemorrhage, recovery prompt. Administered the following: Acid tannici, gr. xx..; liquor Dobell, f℥iv., locally; syr. ferri iodid. internally. Return to normal rapid.

CASE VII.—January, 1895. A. K., girl, aged 2 years. This baby was very poorly nourished; child of poor parents. Slight; extreme anemia; diarrhea; mouth breather. Pharynx and vault filled with hypertrophied tissue. All removed under chloroform; no hemorrhage, recovery good. Did well for a time; condition recurred after six months.

CASE VIII.—October, 1893. E. G., girl, aged 8 years; frequent sore throat; cough; short of breath; nightmare; mouth breather. Both tonsils greatly hypertrophied. Removed under ether; no hemorrhage; recovery good. Entire improvement.

In tonsillar hypertrophy alone (Cases I., II. and VIII.) there is simple obstruction to inspired air, whether it comes through nose or mouth. This means too little oxygen within the lungs; it means an arterial blood little better than the venous.

Tissue hypertrophy within the vault occurred alone in Case IV., and diverted respiratory currents from nose to mouth. The mouth, fashioned for mastication, cannot perform the function of the nose. We do not know all that occurs within the nose while the air is passing, but we do know that through the mouth the air reaches the lungs improperly prepared. In this condition there is also impairment of digestion. Mastication is performed hurriedly in a cavity to which fresh germ-laden air is admitted constantly. One cannot masticate properly and breathe through mouth at the same time. There is a nervous desire to empty mouth quickly. Consequently the stomach suffers.

Either of above conditions alone is burden enough for a growing organism. When the two are combined in one individual, (Cases II., V., VI., VII.,) the result is most pernicious. Added to the disturbances of nutrition there is profound nervous irritability. Irritated nerves, fed by poor blood, point to ruin. In each of the eight cases the general irritability was marked; in four of them there was in addition a specially well-marked reflex. In Cases II. and VIII. there was cough; in III. there was cough and headache; in IV., earache. They all disappeared after operating.

The cases quoted are but a few of those met frequently. They are given for emphasis. Many phenomena spring from the conditions mentioned, but all are in the same line and all tend to the same result—the ruin of the individual. They show the mistakes of nature, and they show how those mistakes may be corrected and overcome. The welfare of the state depends upon its people. So long as the child fashions his own manhood in his own way and out of poor material, there will be poor citizens. He needs only care. Give the boy health and proper training, and he becomes a citizen fit to endure life. He is a prophet of the future. It is our fault if the prophesy does not come to pass.

466 Franklin Street.

---

Hysterioclasic Zones.—Dr. Clozier, of Paris, says that in hysterical patients strong pressure over the apex of the heart applied for about thirty seconds sometimes stops attacks of hystero-epilepsy, hallucinatory conditions and nervous cough.—*North American Practitioner*.

